# Port d’Entrée for Respiratory Infections – Does the Influenza A Virus Pave the Way for Bacteria?

**DOI:** 10.3389/fmicb.2017.02602

**Published:** 2017-12-21

**Authors:** Nikolai Siemens, Sonja Oehmcke-Hecht, Thomas C. Mettenleiter, Bernd Kreikemeyer, Peter Valentin-Weigand, Sven Hammerschmidt

**Affiliations:** ^1^Department of Molecular Genetics and Infection Biology, Interfaculty Institute for Genetics and Functional Genomics, University of Greifswald, Greifswald, Germany; ^2^Center for Infectious Medicine, Karolinska Institutet, Karolinska University Hospital, Stockholm, Sweden; ^3^Institute of Medical Microbiology, Virology and Hygiene, University Medicine Rostock, Rostock, Germany; ^4^Institute of Molecular Virology and Cell Biology, Friedrich-Loeffler-Institute, Federal Research Institute for Animal Health, Greifswald-Insel Riems, Germany; ^5^Center for Infection Medicine, Institute for Microbiology, University of Veterinary Medicine Hannover, Hannover, Germany

**Keywords:** pneumonia, co-infections, Influenza A virus, Gram-positive bacteria, *Streptococcus pneumoniae*, *Staphylococcus aureus*, *Streptococcus pyogenes*, animal models

## Abstract

Bacterial and viral co-infections of the respiratory tract are life-threatening and present a global burden to the global community. *Staphylococcus aureus*, *Streptococcus pneumoniae*, and *Streptococcus pyogenes* are frequent colonizers of the upper respiratory tract. Imbalances through acquisition of seasonal viruses, e.g., Influenza A virus, can lead to bacterial dissemination to the lower respiratory tract, which in turn can result in severe pneumonia. In this review, we summarize the current knowledge about bacterial and viral co-infections of the respiratory tract and focus on potential experimental models suitable for mimicking this disease. Transmission of IAV and pneumonia is mainly modeled by mouse infection. Few studies utilizing ferrets, rats, guinea pigs, rabbits, and non-human primates are also available. The knowledge gained from these studies led to important discoveries and advances in understanding these infectious diseases. Nevertheless, mouse and other infection models have limitations, especially in translation of the discoveries to humans. Here, we suggest the use of human engineered lung tissue, human *ex vivo* lung tissue, and porcine models to study respiratory co-infections, which might contribute to a greater translation of the results to humans and improve both, animal and human health.

## Introduction

In recent years the human microbiota is more and more recognized to play a crucial role in pathogenesis of many diseases (Weinstock, 2012). The upper respiratory tract is a natural niche for potentially pathogenic bacteria embedded in commensal communities forming the nasopharyngeal microbiome. In particular, the microbial communities of the nasopharynx (Hilty et al., 2012) are associated with respiratory diseases, i.e., severe pneumonia, which are responsible for substantial mortality and morbidity in humans worldwide (Prina et al., 2016). The composition of the nasopharyngeal microbiome is highly dynamic (Biesbroek et al., 2014a,b,c) and many factors, including environmental and host factors, can affect microbial colonization (Koppen et al., 2015). Recent studies on neonates have shown that the respiratory microbiota develops from initially maternally transmitted mixed flora with predominance of *Streptococcus viridans* species to niche-specific bacterial profiles containing mostly *Staphylococcus aureus* at around 1 week of age (Bosch et al., 2016a). Between 2 weeks and 6 months after birth, the staphylococcal predominance declines and colonization with *Streptococcus pneumoniae* (pneumococci) as a predominant pathobiont emerges (Miller et al., 2011; Bosch et al., 2016a,b). The dynamic microbiome composition is guaranteed through the interplay between bacterial species, other microbes, and changing environmental conditions, as well as host–bacteria interactions (Blaser and Falkow, 2009). Most of the time, the microbiome and its interplay with the human host are believed to be beneficial for both (Pettigrew et al., 2008; Murphy et al., 2009). However, imbalances in microbial composition can lead to acquisition of new viral or bacterial species and invasion of potential pathogens, which in turn can become detrimental, especially in elderly people and children with an exhausted or immature immune system (Pettigrew et al., 2008; Blaser and Falkow, 2009; Murphy et al., 2009).

One particular example showing imbalances introduced by single dosage of antibiotics was demonstrated by Ichinohe and colleagues (Ichinohe et al., 2011). While commensal respiratory microbiota facilitated immune-support against Influenza A virus infection (IAV), oral treatment with antibiotics resulted not only in a shift of bacterial composition, but also in impaired CD4 T-, CD8 T-, and B-cell immunity following infection with IAV in mice (Ichinohe et al., 2011). Analyses of human oropharyngeal microbiomes during the 2009 H1N1 IAV pandemic revealed that at the phylum level, the abundance of Fermicutes and Proteobacteria was augmented in pneumonia patients as compared to healthy controls (Leung et al., 2013). However, another study published in the same year contradicted these results (Chaban et al., 2013). Chaban and colleagues analyzed microbiomes of 65 patients from H1N1 IAV outbreak in 2009. Although the phylogenetic composition of pneumonia patients was dominated by Fermicutes, Proteobacteria, and Actinobacteria, no significant differences between the patients and healthy controls or any other variables tested, including age and gender, were observed (Chaban et al., 2013).

In this review we discuss secondary bacterial infections of the respiratory tract after primary infection by IAV with a focus on mechanisms by which these interactions are potentially mediated, and we will provide insight into the host contribution and immunological consequences. We further focus on potential animal models suitable for mimicking asymptomatic bacterial colonization and disease progression and thus, enabling to study adaptation strategies, viral-bacterial interactions, and immune responses in these highly lethal co-infections.

## Influenza A Viruses and Pandemics

Influenza A viruses belong to the family of *Orthomyxoviridae* and based on the antigenicity of their haemagglutinin (HA) and neuraminidase (NA) they are classified into 16 classical HA and 9 classical NA subtypes (Neumann et al., 2009). The 8-segmented genomes of influenza A viruses are characterized by a significant plasticity. Due to point mutations and re-assortment events new variants or strains with epidemic or pandemic potential emerge (Neumann et al., 2009). In addition, influenza can be transmitted between animals, including swine, birds, horses, and humans, making it a zoonotic disease (van der Meer et al., 2010). Seasonal influenza usually resolves without consequences in healthy individuals. However, it is estimated that seasonal influenza effects 5–10% of the world’s population resulting in about 250,000 to 500,000 deaths annually (Tjon-Kon-Fat et al., 2016). At greater risk to develop secondary bacterial pneumonia are individuals with comorbidities, elderly people (age > 65), pregnant women, and children under the age of one (Rothberg et al., 2008).

For a long time it was considered that the H1N1 strain, an avian-like H1N1 virus, directly caused most of the fatalities during the 1918–1919 pandemic (Spanish Flu), often from a hemorrhagic pneumonitis rapidly progressing to acute respiratory distress syndrome and death (Osterholm, 2005; Gerberding, 2006; Oxford et al., 2006). The pandemic killed around 50 million people worldwide and remains unique in its severity compared to other big outbreaks. However, many of the findings have been reinterpreted in recent years (Brundage and Shanks, 2007; Chien et al., 2009). It is estimated that around 95% of all severe cases and deaths were attributed to secondary infections with bacterial pathogens, most predominantly by *Streptococcus pneumoniae* (Morens et al., 2008). Individual studies limited to certain regions identified also other pathogens commonly colonizing the respiratory tract, including *Staphylococcus aureus*, group A streptococcus (GAS) and *Haemophilus influenzae* (Brundage and Shanks, 2008). During the next two pandemics (H2N2 Asian Flu 1957-1958 and H3N2 Hong Kong Flu 1968-1969) bacterial co-infections were less likely the cause of death compared to the Spanish Flu (Giles and Shuttleworth, 1957; Trotter et al., 1959). Still, pneumonia accounted for about 44% of deaths during the Asian Flu (Giles and Shuttleworth, 1957). Most fatalities resulting from pneumonia occurred in individuals with chronic conditions, i.e., chronic lung diseases, rheumatic carditis, and hypertension (Giles and Shuttleworth, 1957). In 1957–1958, *S. aureus* was predominantly isolated from fatal pneumonia cases (Hers et al., 1957, 1958; Robertson et al., 1958; Martin et al., 1959), whereas *S. pneumoniae* returned as predominant cause of severe pneumonia during the Hong Kong Flu (Sharrar, 1969; Bisno et al., 1971; Burk et al., 1971; Schwarzmann et al., 1971). Forty years later in 2009, a novel H1N1 virus of swine origin emerged and caused again a pandemic (Dawood et al., 2009, 2012). In contrast to Asian and Hong Kong Flu, mortality rates were rather low, but most deaths occurred in healthy young individuals with no underlying conditions (Reichert et al., 2010; Monsalvo et al., 2011; Dawood et al., 2012). About 25–50% of severe or fatal cases were linked to complications due to bacterial pneumonia (Dominguez-Cherit et al., 2009; Estenssoro et al., 2010; Mauad et al., 2010; Shieh et al., 2010). Although regional variations occurred, pneumococci and *S. aureus* were the most frequently isolated bacterial species (Mauad et al., 2010; Shieh et al., 2010; Rice et al., 2012). Group A streptococcus was absent in many local pneumonia outbreaks associated with viruses, but was predominant in others (Brundage and Shanks, 2008; Ampofo et al., 2010). When it does appear, it is typically third in incidence (Chaussee et al., 2011). Overall, data on pandemic outbreaks suggest that disease severity and mortality can be linked to secondary bacterial pathogens with variations depending on regions and state of immunity of the population (Brundage and Shanks, 2008; Shanks et al., 2010, 2011; McCullers, 2013).

## Gram-Positive Bacteria Associated With Respiratory Infections

**FIGURE 1:**
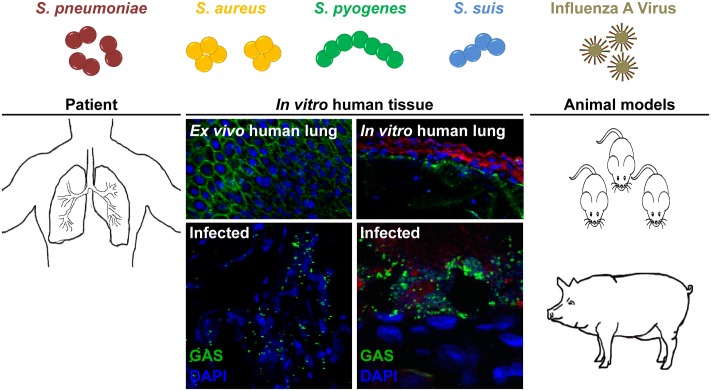
Potential models to study bacterial and viral co-infections of the respiratory tract. *S. pneumoniae, S. aureus, S. pyogenes*, and *S. suis* are frequent colonizers of the upper respiratory tract. Seasonal IAV infection can lead to an increased risk of secondary bacterial infections, i.e., pneumonia. Several experimental models can be used for studying these severe infections. Patient samples, including *ex vivo* lung tissue are materials of choice, but they are rare due to ethical considerations. Tissue engineering approaches closely resemble the 3D architecture, cellular composition, and matrix complexity of the respective organ and were proven as useful tool to study infectious diseases. *In vivo* bacterial and viral co-infections are mainly performed in mice, which does not necessarily resemble the human physiology and immune system. Thus, we suggest using the porcine model, which nearly resembles over 80% of the human immune system.

There is increasing evidence that the nasopharyngeal microbiota plays an important role in the pathogenesis of acute viral respiratory infections (Teo et al., 2015; de Steenhuijsen Piters et al., 2016; Rosas-Salazar et al., 2016a,b). Respiratory viruses, including IAV, have been shown to alter bacterial adherence and colonization leading to an increased risk of secondary bacterial infections (Tregoning and Schwarze, 2010). Pneumococci, *S. aureus*, and GAS are important human Gram-positive pathogens. All of them are frequent colonizers of the human nasopharynx and they share many features including pathogenic mechanisms and clinical aspects (**Figure 1**). However, they also have unique properties.

*Staphylococcus aureus* colonizes persistently about 30% of the human population and typical niches include nares, axillae, and skin (Peacock et al., 2001; von Eiff et al., 2001; van Belkum et al., 2009). They cause a variety of clinical manifestations ranging from mild skin infections to fatal necrotizing pneumonia. In the last decades, the pathogen became resistant to an increasing number of antibiotics and methicillin-resistant *S. aureus* (MRSA) is now a major cause of hospital acquired infections (Hartman and Tomasz, 1984; Ubukata et al., 1989; Zetola et al., 2005). Also the rise of community-acquired *S. aureus* strains is of special concern, because certain clones are associated with very severe infections (Rasigade et al., 2010). Recent prospective studies demonstrated an increase in proportion of community-acquired methicillin-sensitive *S. aureus* in severe pneumonia cases (McCaskill et al., 2007; Sicot et al., 2013).

The pneumococcus is a typical colonizer of the human nasopharynx. About 20–50% of healthy children and 8–30% of healthy adults are asymptomatically colonized (McCullers, 2006). Pneumococci cause diseases ranging from mild, i.e., sinusitis, conjunctivitis, and otitis media, to more severe and potentially life-threatening infections, including community-acquired pneumonia, bacteraemia, and meningitis (Bogaert et al., 2004; Valles et al., 2016). This bacterium is associated with high morbidity and mortality rates in risk groups such as immunocompromised individuals, children, and elderly (Black et al., 2010; Valles et al., 2016).

Group A streptococci colonize the mouth and upper respiratory tract in about 2–5% of world’s population (Okumura and Nizet, 2014). The most common, non-invasive and mild infections caused by GAS are tonsillitis and pharyngitis with estimated 600 million cases per year (Carapetis et al., 2005). Listed as number nine in the list of global killers with around 500,000 deaths annually (Carapetis et al., 2005), it is obvious that this pathogen can cause severe invasive infections, including pneumonia, sepsis, streptococcal toxic shock syndrome, and necrotizing skin infections (Cunningham, 2000; Carapetis et al., 2005).

Although all three pathogens are able to cause highly lethal diseases, the most fatal remains the pneumococcus, estimated to cause ca. 10% of all deaths in children below 5 years of age (O’Brien et al., 2009), in the elderly (Marrie et al., 2017), and in immuno-compromised individuals (Baxter et al., 2016).

## IAV Induced Lung Tissue Inflammation and Damage Trigger Subsequent Bacterial Infection

### Initial Steps of Bacterial and Viral Co-infections

**FIGURE 2:**
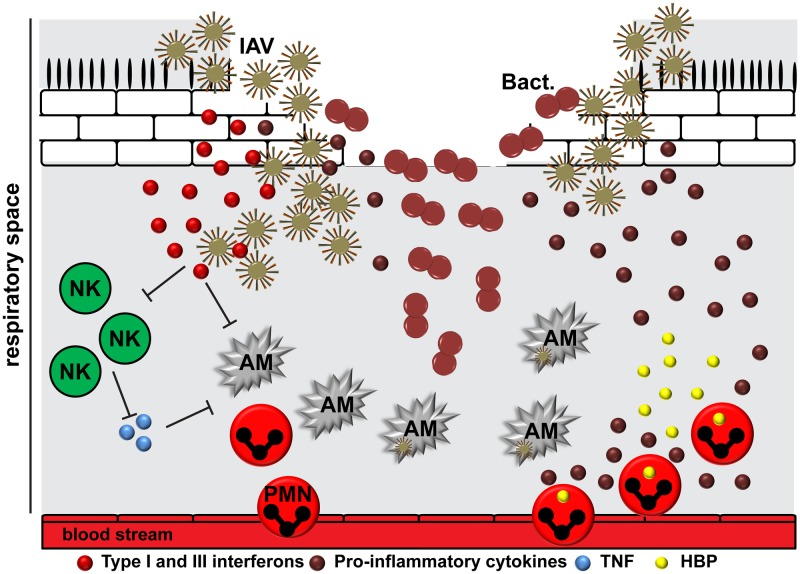
The interplay between IAV, bacteria, and the human host. The epithelial damage due to viral replication provides a beneficial environment for bacterial (Bact.) attachment. IAV is able to induce suppression and killing of resident alveolar macrophages (AM), which in turn delays viral clearance. The release of viral RNA activates different immune response pathways resulting in cytokine storm. Type I and III interferons compromise the immune recognition of Gram-positive bacteria by neutrophils and macrophages. In addition, they might suppress natural killer cell function (NK), including release of TNF, which activates alveolar macrophages. After initial inflammation, the situation might worsen due to cellular infiltration of the lungs by neutrophils (PMN), leading to an increased degranulation and tissue damage by effector molecules, including heparin-binding protein (HBP).

Influenza A virus binds via HA to either α2,3- or α2,6-linked sialic acid at the surface of epithelial cells of the upper and lower respiratory tract (Webster et al., 1992). Seasonal strains show usually affinity to α2,6-linked sialic acids that are expressed in the human trachea, whereas avian-like viruses preferentially bind to α2,3-linked sialic acids of alveolar type II cells (Shinya et al., 2006; van Riel et al., 2007, 2010). The release of viral genomic RNA into the cytosol activates different immune response pathways. Binding of viral RNA to retinoic acid inducible gene 1 induces the expression of type I and III interferons and activates transcription factor NF-κB, which in turn activates the release of pro-inflammatory cytokines (Durbin et al., 2013; Iwasaki and Pillai, 2014). In addition, inflammasome activation leads to the release of IL-1β and IL-18 (Pothlichet et al., 2013; Iwasaki and Pillai, 2014). All these responses are supposed to promote viral clearance. However, the presence of viral proteins during infection induces also direct activation of the intrinsic or indirectly the activation of the extrinsic apoptotic pathway via production of inflammatory cytokines, resulting in apoptosis or even necrosis of the epithelium (Korteweg and Gu, 2008). Furthermore, aberrant coagulation induced by virus infection causes a hyper-inflammatory response (Yang and Tang, 2016). All these events contribute to lung tissue injury (Imai et al., 2008; Davidson et al., 2014). The epithelial damage due to viral replication provides a beneficial environment for initial bacterial attachment (Plotkowski et al., 1993). On the other hand, already colonized bacteria might enhance influenza virus virulence either by directly secreting proteases that cleave and activate HA (**Figure 2**) (Bottcher-Friebertshauser et al., 2013) or, indirectly, by activating host proteases such as plasminogen, which increases replication rates and infectivity of the virus (Scheiblauer et al., 1992; Tse and Whittaker, 2015).

Potentially pathogenic bacteria, including the three species mentioned above, express an arsenal of virulence factors responsible for attachment to human host structures. Microbial surface components recognizing adhesive matrix molecules (MSCRAMMs), such as PspC, PspA, and PsaA in pneumococci (Hammerschmidt, 2006), SPA, FnbA, ClfA, and ClfB in *S. aureus* (Bartlett and Hulten, 2010; Otto, 2010), and M-protein, PrtF1, and PrtF2 in GAS (Cunningham, 2000), respectively, and so-called moon-lightning proteins expressed by all three species, e.g., GAPDH, enolase or PGK (Fulde et al., 2013), enable the bacteria to attach to damaged cells or molecules of the extracellular matrix, including fibronectin, fibrin, fibrinogen, and collagens, or fibrinolytic proteins like plasminogen (McCullers and Rehg, 2002; Bergmann and Hammerschmidt, 2007; Linke et al., 2012; Siemens et al., 2012; Voss et al., 2012). Once the initial attachment occurs, bacterial cytotoxins including pneumolysin of pneumococci (Garcia-Suarez Mdel et al., 2007; Zahlten et al., 2015), α-hemolysin and leukocidins of *S. aureus* (Mairpady Shambat et al., 2015), and Streptolysins S and O and Streptococcal pyrogenic exotoxin B of *S. pyogenes* (Tsai et al., 1998; Gurel et al., 2013; Siemens et al., 2015, 2016), can synergize with viral counterparts to further increase lung tissue pathology. Additional potential mechanisms by which the initial colonization of the lower respiratory tract and lung tissue damage might occur include potentiation of the development of pneumonia by IAV neuraminidase through enzymatic removal of sialic acid from the lung, thus exposing host receptors for pneumococcal adherence (McCullers and Bartmess, 2003). The host inflammatory state in response to viral infection can alter presentation of receptors on the surface, thus allowing bacterial invasion (Cundell and Tuomanen, 1994). As the patient begins to recover from viral infection, secondary bacterial infections might occur (Louria et al., 1959) due to the incomplete wound healing and exposure of host membrane components, including laminin, collagens type I and IV to classical bacterial MSCRAMMs (Louria et al., 1959; Puchelle et al., 2006).

### Immune Modulation in Bacterial and Viral Co-infections

Epithelial cells are the first responders to infections in the lung, followed by the tissue resident alveolar macrophages. They promote viral clearance via phagocytosis, efferocytosis, and release of cytokines and chemokines to promote immune responses (Hashimoto et al., 2007; Kumagai et al., 2007; Wang et al., 2012; Hillaire et al., 2013). Respiratory viruses like IAV are able to induce suppression and killing of the resident alveolar macrophages (**Figure 2**) (Ghoneim et al., 2013). These cells are usually replaced by differentiation of recruited blood derived monocytes into macrophages of different polarization patterns. This in turn creates a delay in pathogen clearance and opens a window for host susceptibility to secondary bacterial infections, colloquially named superinfections (Ghoneim et al., 2013). In addition, induction of interferons as a response to viral infection compromises the immune sensing of Gram-positive bacteria by neutrophils and macrophages, which would normally clear the bacteria from the lungs (**Figure 2**) (Sun and Metzger, 2008; Tian et al., 2012). The exact mechanism underlying this phenomenon is still not understood. Several studies suggested that viral RNA activates Toll-like receptors (TLR) 2 and TLR4 and, consequently, the production of type I interferons to promote an antiviral state (Shahangian et al., 2009). The subsequent infection with Gram-positive bacteria, e.g., pneumococci, enhances the type I interferon expression, which in turn suppresses production of the CCL2 chemokine and recruitment of macrophages (Nakamura et al., 2011). Another study by Shahangian et al. (2009) revealed that the antiviral state leads to impaired production of neutrophil chemoattractants CXCL1 and CXCL2, which in turn promotes less effective immune responses due to attenuated neutrophil functions during the early phase of pneumococcal invasion. Other studies found that IAV exposed lungs had impaired natural killer (NK) cell responses in the airway to subsequent *S. aureus* infection (Small et al., 2010). Reduced TNFα production by NK cells was identified as a crucial upstream mechanism of depressed antimicrobial activities by alveolar macrophages (**Figure 2**) (Small et al., 2010). It seems likely that IAV NA is also able to activate host cell receptors in a TGF-β dependent manner, which in turn promotes GAS invasion and subsequent lung pathology (Li et al., 2015). *In vitro* studies on the interplay between IAV-pneumococci and human dendritic cells revealed TLR3 as a crucial sensor of viral and bacterial RNA leading to enhanced IL-12p70 production, which in turn might promote an anti-viral state by upregulation of interferons (Yamamoto et al., 2004; Spelmink et al., 2016). However, it should be noted that depending on the bacterial species the disease manifestation and underlying innate immune responses might vary (Sharma-Chawla et al., 2016).

A lot of the experimental studies on disease mechanisms and immune responses are based on a subsequent bacterial infection within hours or a few days post IAV infection. However, bacterial infiltrations of the lungs might occur much later, i.e., during the onset of wound healing after partial clearance of IAV, which has been reported in most studies performed in recent years (Snelgrove et al., 2008; Hussell and Cavanagh, 2009). These processes are characterized by a general anti-inflammatory state and suppression of mechanisms involved in pathogen clearance due to increased interleukin-10 production (van der Sluijs et al., 2004; Metzger and Sun, 2013). The anti-inflammatory state suppresses the expression of pattern recognition receptors (PRR) on professional phagocytes leading to impaired phagocytosis and killing of microbes. These events might allow bacterial overgrowth in the lungs and tissue pathology (Sun and Metzger, 2008; Goulding et al., 2011).

Like other severe infectious diseases caused by single agents, pneumonia is characterized by hyper-inflammatory conditions of the lungs at the onset of infection followed by a hypo-inflammatory state with immune paralysis (Morton et al., 2014). In co-infections, after initial inflammation in response to viral infection the situation might worsen due to bacterial invasion and enhanced cellular infiltration of the lungs by neutrophils, leading to an increased tissue damage and cytokine storm (**Figure 2**) (Conenello et al., 2007; McAuley et al., 2007, 2010; Porto and Stein, 2016). Furthermore, the coagulation system becomes activated and contributes to the pathophysiological response to infection (van der Poll and Herwald, 2014). Bacteria like pneumococci, *S. aureus*, and GAS can activate and modulate the coagulation system, leading to extensive expression of tissue factor and increasing the risk of severe coagulopathy (Nguyen et al., 2012; Shannon et al., 2013; Walters et al., 2016).

Bacterial pathogens also express a variety of cytolytic toxins that can contribute to inflammation and tissue pathology. Pneumolysin, a pneumococcal pore-forming toxin with low affinity to lung epithelial cells, can damage neutrophils by utilizing P2X7 receptor (Domon et al., 2016). Staphylococcal cytotoxins (α-toxin and leukocidins, including Panton-Valentine leucocidin, PVL) are associated with severe tissue pathology, strong upregulation of chemokines, and increased neutrophil influx of the lungs (Mairpady Shambat et al., 2015). GAS toxins, including SLO and SpeB, are capable of directly causing tissue damage and promoting pro-inflammatory states through neutrophil lysis (Snall et al., 2016; Uhlmann et al., 2016). The cytolytic effects caused by bacterial toxins might synergize with the outcome of IAV cytotoxic accessory protein, PB1-F2, mediated tissue pathology leading to enhanced cytokine production (Ramos and Fernandez-Sesma, 2012). Taken together, most likely synergistic effects of the pathways that are involved in bacterial and viral inflammation lead to enhanced immune activation and higher morbidity and mortality (Joyce et al., 2009; Koppe et al., 2012; Ramos and Fernandez-Sesma, 2012; Bucasas et al., 2013; Kuri et al., 2013). **Figure 2** summarizes the interplay between virus, bacteria, and host.

## Suitable *In Vivo* Models for Mimicking Respiratory Infections

### Mouse Models

Experimental animal models are a useful tool to study *in vivo* effects of different infectious agents and they represent approximately 3% of all pneumonia research published in peer-review journals (Hraiech et al., 2015). However, the constant increase of animal studies in the last decades is in contrast to their reproducibility in humans (Hackam and Redelmeier, 2006). Hackam and colleagues identified 2,000 articles published between 1980 and 2006 in seven leading scientific journals that regularly publish animal studies (Hackam and Redelmeier, 2006). Seventy-six out of 2,000 were highly cited with a median citation count of 889. Out of these 76 studies 28 were replicated in human randomized trials, 14 were contradicted, and 34 remained untested (Hackam and Redelmeier, 2006). Only 1.4% of the animal studies published in high-impact journals were translated in human randomized trials (Hackam and Redelmeier, 2006), whereas about 44% replication rate was reported for highly cited human studies (Ioannidis, 2005). In pneumonia models, mammalians are mostly used because of their anatomical and physiological proximity to humans (Hraiech et al., 2015). To monitor extensive physiological studies, larger mammalian species, including ferrets, dogs, rabbits, pigs, and baboons are the models of choice (Mizgerd and Skerrett, 2008). However, rodents and in particular mice are used more frequently as a pneumonia model organisms. Rapid reproductive rate, small size, less complicated handling, the ability to reproduce and compare results with already published bacterial and viral mono-infections, detailed knowledge of genetics and immune responses, and a plethora of available reagents to study infections in mice are reasons for the use of these animals. To avoid variations in responses due to genetic diversity inbred mice strains are useful tools for studies aiming to elucidate molecular mechanisms of diseases. In addition, genetic engineering allowed to generate a wide variety of mouse variants with gain-of-function, loss-of-function or reporter genes (Mizgerd and Skerrett, 2008).

As outlined above, many *in vivo* mice studies on bacterial and viral co-infections provided useful insights into severe pneumonia, including (i) the fact that viral infection primes the host for bacterial susceptibility leading to severe secondary infection (Hashimoto et al., 2007; Shahangian et al., 2009; Chaussee et al., 2011; Nakamura et al., 2011), (ii) pathogen synergism (Tsai et al., 1998; McCullers and Rehg, 2002; Garcia-Suarez Mdel et al., 2007; Gurel et al., 2013; Mairpady Shambat et al., 2015; Zahlten et al., 2015), (iii) enhanced inflammatory response at the onset of infection (Korteweg and Gu, 2008; Durbin et al., 2013; Pothlichet et al., 2013; Iwasaki and Pillai, 2014) leading to increased alveolar damage followed by immune paralysis with defective clearance of microorganisms (Shinya et al., 2006; van Riel et al., 2007, 2010), and (iv) host receptor availability for sustained bacterial infection (Louria et al., 1959; Plotkowski et al., 1993; Cundell and Tuomanen, 1994; Puchelle et al., 2006; Korteweg and Gu, 2008). However, mouse models for bacterial and/or viral infections have several limitations. Most of the bacterial and viral species under study are human pathogens. In recent years it was also shown that host genetic variations and sex differences have an impact on predisposition, severity, and outcome of infection (Chella Krishnan et al., 2015, 2016) While C57BL/6 and BALB/c mice are characterized by a higher resistance, DBA/2 strains are more susceptible and permissive to bacterial and viral strains (Alymova et al., 2011; Chella Krishnan et al., 2015, 2016). In addition, transmission of IAV and bacteria is inefficient in adult mice, thus requiring alternative animal models, including neonatal mice or ferrets (Diavatopoulos et al., 2010; McCullers et al., 2010). IAV was shown to be essential for pneumococcal transmission from colonized mice to their naive littermates and the transmission occurred only when all mice were infected with IAV (Diavatopoulos et al., 2010).

### Ferret Models

The facilitated transmission of pneumococci after IAV infection was confirmed by Mc Cullers et al. in ferrets (*Mustela putorius furo*) (McCullers et al., 2010). The pneumococcal disease manifestation and transmission between animals was enhanced if animals had previously been infected with IAV (McCullers et al., 2010). Ferrets are naturally susceptible to IAV isolated from different species, including humans, birds, and swine (Thangavel and Bouvier, 2014). The infection of ferrets with human seasonal IAV isolates results in an upper respiratory tract infection similar to human influenza infection (Tripp and Tompkins, 2009). In contrast to mice, non-adapted human IAV can be used for the infection. Unfortunately, there are only few reports on bacterial and IAV co-infections in this model organism. A report by Sanford and Ramsay showed enhanced staphylococcal colonization of the upper respiratory tract in IAV infected animals as compared to non-infected, while no difference between both groups was observed in group B streptococcal infection (Sanford and Ramsay, 1987). In contrast, Smith and Mc Cullers reported lack of establishment of staphylococcal infection even when ferrets were pre-infected with IAV (Smith and McCullers, 2014). The biggest advantages of using ferrets as a model include (i) their susceptibility to non-adapted human pathogens, (ii) efficiency in transmitting IAV and bacteria from one individual to another, and (iii) presentation of the clinical signs of disease manifestation akin to human influenza infection. Unfortunately, their limited availability, complex husbandry, and limited accessibility to ferret-specific reagents makes this research difficult to perform (Bouvier and Lowen, 2010).

### Guinea Pig Models

In recent years, the guinea pig (*Cavia porcellus*) was also used in pneumonia research. The physiology and anatomy of the guinea pig lung resembles to a certain extent the human lung and this model organism is often used in non-infectious lung diseases, including asthma and chronic obstructive pulmonary disease (Canning and Chou, 2008). In addition, its commercial availability, ease of husbandry, the ability to work with non-adapted pathogens and the efficiency of transmission are reasons for using this *in vivo* model (Bouvier and Lowen, 2010). Guinea pigs are susceptible to human, avian, and swine influenza viruses. Although viral replication can be readily detected upon intranasal inoculation in the upper respiratory tract and the lungs, guinea pigs exhibit only minor clinical symptoms (Lowen et al., 2006; Gabbard et al., 2014). However, the lung pathology of human IAV infected guinea pigs correlates with the clinical severity of human infection (Gabbard et al., 2014). Transmission of pneumococci in guinea pigs is promoted by co-infection with Sendai virus (Saito et al., 1988). Guinea pigs infected with pneumococci alone and cage-mated with non-treated contact animals transmitted the bacteria only in 7% of cases, while Sendai-virus infected, co-housed guinea pigs acquired pneumococcal infection in 83% of contacts (Saito et al., 1988). Another study evaluated antibiotic efficacy in invasive pulmonary infection caused by penicillin resistant pneumococcus (Ponte et al., 1996). Intra-tracheal instillation of 3 × 10^9^ CFU of *S. pneumoniae* induced a fatal pneumonia and bacteremia in 85% of untreated animals within 46 h (Ponte et al., 1996). As with ferrets, there is a paucity of data describing immune responses to pulmonary infectious agents. This is in parts due to the lack of species specific reagents, which is a disadvantage in using this model organism.

### Rat Models

Recently, the cotton rat (*Sigmodon hispidus*) was reported to be susceptible to IAV. Nasal and pulmonary infection in adult inbred cotton rats did not require viral adaptation (Ottolini et al., 2005). The infection led to increased breathing rates accompanied by weight loss and decreased body temperature. Replication of IAV was more extensive in nasal tissues than the lung, and persisted for six consecutive days. Tissue pathology included damage of bronchiolar epithelium and the animals developed pneumonia which persisted for nearly 3 weeks (Ottolini et al., 2005). In bacteriological studies rats are more frequently used. There are numerous rat models investigating the impact of diabetes (Oliveira et al., 2016), metabolic syndromes (Feng et al., 2015), cirrhosis (Preheim et al., 1991), pharmaco-kinetics and dynamics (Antonopoulou et al., 2015; Hoover et al., 2015), intoxication (Davis et al., 1991), immunization (Iinuma and Okinaga, 1989), and general bacterial virulence factors (Shanley et al., 1996) on development of pneumococcal, streptococcal, and staphylococcal pneumonia and lung pathology. Unfortunately, there are only few studies on bacterial and viral co-infections in rats. The first was performed by Harford et al., 1946 (Harford et al., 1946). The authors concluded that the secondary bacterial pneumonia does not convert the sub-lethal viral infection to a lethal outcome (Harford et al., 1946). Another study on human respiratory syncytial virus and *S. pneumoniae* revealed that rats were easily colonized with pneumococci, but viral replication after subsequent infection was strain dependent. In addition, neither pneumococci nor the virus spread from the upper to the lower respiratory tract, and neither pathogen was transmitted to naive cage mates (Nguyen et al., 2015). Although rats share a lot of immune features with humans, including nitric oxide production by macrophages (Carsillo et al., 2009), the biggest disadvantages are low animal availability, aggressiveness of the species, and the lack of specific reagents.

### Rabbit Models

Rabbits (*Oryctolagus cuniculus*) are well known for their use in studying cardiovascular diseases, antibody production, and eye research. Rabbits were also employed to study pneumonia, although only a few models are available. Typical read-out parameters include survival, leukocyte infiltration of the lungs, lung pathology, and assessment of drug concentration in serum. One of the first studies on pneumococcal pneumonia in rabbits was performed in Kline and Winternitz (1913). This study revealed that rabbits possess an active immunity if they have recovered from one attack of experimental pneumonia and they may subsequently resist repeated intra-tracheal dosages of pneumococci (Kline and Winternitz, 1913). In 1926 an infection by inhalation of Type I pneumococci was established in rabbits (Stillman and Branch, 1926). The bacteria infiltrated easily the lower respiratory tract and pneumococci which reached the lungs usually disappeared within hours and fatal septicemia appeared in some of the animals (Stillman and Branch, 1926). Most recent rabbit models of pneumococcal and staphylococcal pneumonia are based on intra-bronchial or intra-pulmonary infections which make them useful for pathogenesis (Diep et al., 2010, 2017), as well as drug efficiency and efficacy studies (Cabellos et al., 1992; Croisier-Bertin et al., 2011). However, this infection route requires surgery and species-specific reagents are scarce. In IAV research rabbits are frequently used for antibody production and for studies on antibody kinetics following single or multiple IAV administrations (Loza-Tulimowska et al., 1977). Also, rabbits are used for safety investigations of vaccines (e.g., CoVaccine HT or Aflunov) (Heldens et al., 2010; Gasparini et al., 2012). In recent years the shedding of avian IAV by cottontails (*Sylvilagus* spp.) was investigated revealing that nasally and orally inoculated cottontails shed relatively large quantities of viral RNA (Root et al., 2014). Notably, low viral titers were found to be sufficient to initiate viral replication in cottontails (Root et al., 2017). However, despite their susceptibility to IAV infection, rabbits are only rarely used as model for IAV pathogenesis since they offer no improvement over other established infection models.

### Non-human Primate Models

Macaques represent the major non-human primate for studying infectious diseases. They are omnivorous and adaptable. The species most commonly used are rhesus macaques (*Macaca mulatta*) and cynomolgus macaques (*Macaca fasciluraris*). Although it was shown early that macaques were susceptible to IAV (Saslaw et al., 1946), the animal models of choice remained ferrets and mice. Recently, macaques have been used to compare the pathogenesis of highly virulent 1918 pandemic IAV and the pathogenic bird flu strain (H5N1) with a conventional H1N1 strain (Rimmelzwaan et al., 2001). Cynomolgus macaques infected with highly pathogenic H5N1 developed acute respiratory distress syndrome, fever, and necrotizing pneumonia (Rimmelzwaan et al., 2001). The 1918 IAV strain induced dysregulation of the antiviral response leading to insufficient protection of the host, which in turn resulted in acute respiratory distress and a fatal outcome (Kobasa et al., 2007). The 2009 pandemic H1N1 US isolate caused severe pathological lesions in the lungs of the macaques (Itoh et al., 2009). The three studies mentioned above used combined intra-tracheal delivery of high doses of virus. A recent study by Marriott et al. analyzed the outcome of challenge routes, including inhaled aerosol and intra-nasal instillation with low to moderate doses of H1N1 in cynomolgus macaques (Marriott et al., 2016). Virus replication was detected in all challenge groups, although the disease remained sub-clinical.

In bacteriological studies non-human primates are rarely used. For group A streptococcal infection longitudinal transcriptome analyses were performed in experimental pharyngitis (Virtaneva et al., 2005) and lower respiratory tract infection in cynomolgus macaques (Olsen et al., 2010a). The lower respiratory tract disease observed in macaques after GAS infection mimicked the clinical and pathological features of severe bronchopneumonia in humans (Olsen et al., 2010a). Another study by Olsen and colleagues analyzed the contribution of PVL of a highly virulent USA300 *S. aureus* strain in respiratory infection (Olsen et al., 2010b). Although the lower respiratory tract disease observed in monkey mimicked the clinical and pathological features of early mild to moderate pneumonia in humans, no involvement of PVL in lung pathology or immune cell influx of the lungs could be detected (Olsen et al., 2010b). The same research group has developed a non-lethal IAV (H3N2)-*S. aureus* co-infection model in cynomolgus macaques (Kobayashi et al., 2013). Pneumonia progression was monitored by clinical parameters assessment, blood chemistry, nasal swabs, and pathology of the lungs. Seasonal IAV infection in healthy cynomolgus macaques caused mild pneumonia, but did not predispose the animals to subsequent severe infection with the USA300 clone (Kobayashi et al., 2013).

Although macaques are frequently used for evaluation of pneumococcal vaccine efficacy, including testing the impact of 13-valent pneumococcal conjugate vaccine and 23-valent pneumococcal polysaccharide vaccine on antigen-specific memory B cell repertoires (Jia et al., 2017), only two studies on pneumococcal carriage and pneumonia were conducted in the last decade. In 2013, Philipp and colleagues analyzed the carriage rate of pneumococcus in 158 colony animals. None of the surveyed rhesus macaques carried *S. pneumoniae* in the nasopharynx (Philipp et al., 2012). The authors concluded that rhesus macaque is probably not a natural host of pneumococci. But, when infants were colonized with 19F strain via nasopharyngeal instillation, the colonization was induced in eight of eight infants, lasted for 2 weeks in all animals and for 7 weeks in more than 60% (Philipp et al., 2012). The same group tested detoxified pneumolysin (dPly) and pneumococcal histidine triad protein D (PhtD) as potential vaccine candidates to prevent pneumonia (Denoel et al., 2011). After immunization the rhesus macaques were challenged with a 19F pneumococcal strain. AS02-adjuvanted PhtD-dPly vaccine protected the animals against *S. pneumoniae*-induced pneumonia, which was linked to the capacity (i) to greatly reduce bacterial load within the first week post-challenge and (ii) the levels of PhtD- and Ply-specific antibodies (Denoel et al., 2011). Although only a few macaque studies on pneumonia exist, due to the close proximity to humans in terms of physiology and immunity, these animals can be a good model in the context of translational studies evaluating therapeutics and prophylaxis.

### Porcine Models

Despite the wide use of different animal models, the optimal *in vivo* model for human pneumonia remains to be identified. Small mammals including rodents are well known from a biological, genetic, and immunological point of view and are easy to maintain. The choice of these particular animals for infectious disease studies is often a result of a compromise between technical and financial options. However, they are also far from humans’ anatomy, physiology, immunology, and susceptibility to exclusively human pathogens. The experimental animal model should be chosen based on responses comparable to humans. Primates are usually legally reserved to specific topics. In this case, pigs could be an appropriate model system for studying infectious diseases including pneumonia (**Figure 1**). The composition and size of the porcine genome is comparable to that of humans (Hart et al., 2007). In addition, human and porcine organs have many common features and functions (Swindle et al., 2012). The upper respiratory tract of humans and pigs, including the lymphoid tissue in the nasopharynx, is anatomically similar. Furthermore, like humans, pigs possess tonsils, which are absent in mice (Horter et al., 2003). A major advantage of studying infectious diseases by utilizing pigs as a host organism is that pigs have a full set of innate and adaptive immune effectors. According to whole genome sequencing results the porcine immune system resembles over 80% of the human immune system, whereas mice share less than 10% with humans (Dawson et al., 2016). Most of the immune cell compartments identified in humans are also present in pigs (Piriou-Guzylack and Salmon, 2008; Fairbairn et al., 2011). In contrast to mice and similar to humans, pigs have 50–70% of circulating polymorph nuclear cells (Fairbairn et al., 2011). In addition, all functional cytokines or orthologs involved in Th1, Th2, Th17, and Treg paradigm and corresponding immune cells have been described in pigs (Murtaugh et al., 2009; Kaser et al., 2011; Kiros et al., 2011). Especially the very prominent human pro-inflammatory chemo-attractant, CXCL8, is present as an ortholog in pigs, whereas there is no homologue in mice (Fairbairn et al., 2011). In contrast to human monocytes, which can be divided in three subclasses (classical CD14^+^CD16^-^, non-classical CD14^+^CD16^+^, and intermediate CD14^++^CD16^+^), porcine monocytes consist of four subclasses (Chamorro et al., 2005; Fairbairn et al., 2013). Like human monocytes they express adhesion molecules, such as VLA-4 and LFA-1 and co-stimulatory molecules, including CD80 and CD86 (Chamorro et al., 2005).

The pig has previously been used to mimic a number of human infectious diseases. Examples for *S. aureus* infections with this model organism are wound infections (Sanden et al., 1989; Svedman et al., 1989), osteomyelitis (Jensen et al., 2010), and sepsis (Nielsen et al., 2009). Intravenous inoculation of piglets with pneumococci led to bacteremia during a 5 days period and was associated with fever and septic arthritis. Intranasal inoculation of piglets led to colonization for at least six consecutive days without causing clinical signs (De Greeff et al., 2016). In addition, research on respiratory infections of pigs by human pathogens including *S. aureus* (Luna et al., 2009), *Mycobacterium tuberculosis* (Gil et al., 2010), *Bordetella pertussis* (Elahi et al., 2007), *Pseudomonas aeruginosa* (Luna et al., 2009), and IAV (Khatri et al., 2010), was performed in recent years. The fact that pigs and humans are infected with identical subtypes of IAV (H1N1, H3N2), and show similar clinical presentation and pathogenesis, makes pigs an ideal model organism for studies on respiratory co-infections (Van Reeth et al., 1998). Especially IAV infections are already well established in swine (Van Reeth et al., 1998, 2002a,b; Jung et al., 2007; Khatri et al., 2010; Barbe et al., 2011).

In addition to the limited number of publications on pigs and human pathogens, a lot can be translated and learned from studies on the porcine zoonotic pathogen *Streptococcus suis. S. suis* usually inhabits mucosal surfaces of tonsils, nares, genital and alimentary tract of piglets. Once the microbial balance is disturbed, the bacteria can cause meningitis, septicemia, arthritis, and pneumonia in pigs (Staats et al., 1997). Some *S. suis* strains are considered to be hyper-virulent and others hypo- or avirulent. In general, serotype 2 is most frequently isolated from diseased pigs (Staats et al., 1997). *S. suis* can also cause severe diseases in humans including septicemia, meningitis, arthritis, and streptococcal toxic shock syndrome (Tang et al., 2006; Yu et al., 2006; Gottschalk et al., 2007). Although many *in vivo* studies on *S. suis* have been performed by utilizing mice as a model organism (Seitz et al., 2012; Auger et al., 2016), several other studies have shown the advantage of using swine as a natural host for *S. suis* (Bi et al., 2014; Ferrando et al., 2015). A recent publication by Lin and colleagues on H1N1 and *S. suis* co-infected piglets demonstrated the synergistic effects of both pathogens (Lin et al., 2015). Co-infected piglets had more severe clinical presentation and pathological changes in the lung, as compared to animals infected with single pathogens (Lin et al., 2015). In addition, genes associated with immune responses, inflammatory cytokine production, and apoptotic pathways were highly overexpressed in the co-infected group (Lin et al., 2015). Although the porcine model seems to be ideal to mimic human infectious diseases, there are also disadvantages, including, e.g., requirement for specialized experimental animal facilities, time consuming management, high maintenance costs, and limited availability of transgenic animals.

## *Ex Vivo* and *In Vitro* Complex Models of Pneumonia

Although the use of animals contributes greatly to our understanding of infectious diseases, human 3D-organotypic tissue models and *ex vivo* organ tissues should be considered, as they are most valuable tools to study host–pathogen interactions in a more complex setting (**Figure 1**). Tissue engineering approaches were originally focused on regenerative medicine (Langer and Vacanti, 1993). In contrast to standard monolayer cell cultures, tissue models much more closely resemble the 3D architecture, cellular composition, and matrix complexity of the respective organ. In recent years tissue engineering was also successfully employed in a number of studies in infectious diseases, including Zika virus infections of cerebral organoids (Lancaster et al., 2013; Dang et al., 2016), *Helicobacter pylori* infections of gastric epithelial organoids (McCracken et al., 2014; Schlaermann et al., 2016), *Escherichia coli* and Rotavirus infections of gastrointestinal and small intestinal enteroids (Saxena et al., 2015; VanDussen et al., 2015), *Entamoeba histolytica* or Hepatitis B virus infections of hepatic sinusoid tissue (Petropolis et al., 2014, 2016), group A and G streptococcal or staphylococcal infections of skin tissue models (Siemens et al., 2015, 2016; Mairpady Shambat et al., 2016), and staphylococcal and Andes hantavirus infections of human lung tissue (Mairpady Shambat et al., 2015; Sundstrom et al., 2016). The adaptability of these tissue-engineered models to multiple pathogens suggests a great potential for studies of infectious diseases. For instance, the lung tissue model relevant for pneumonia consists of lung fibroblasts embedded in a collagen matrix with a stratified epithelial layer on top (Nguyen Hoang et al., 2012). The engineered tissue is suitable for implanting and studying immune cells, including dendritic cells, monocytes, macrophages, and even peripheral blood mononuclear cells (Nguyen Hoang et al., 2012; Mairpady Shambat et al., 2015). A recent publication demonstrated a two-hit-event of lung pathology in staphylococcal necrotizing pneumonia (Mairpady Shambat et al., 2015). While the α-toxin had direct damaging effect on the lung epithelium, PVL induced lung pathology indirectly through the lysis of neutrophils (Mairpady Shambat et al., 2015). All the studies mentioned above highlight a significant progress in the field of infectious diseases not only from a scientific point of view but also by contributing to the three R principle of animal experimentation (Russell, 1995).

On these terms, the use of cultured *ex vivo* human organ biopsies, which are rare due to ethical considerations, is an additional option to study host–pathogen interactions. This *ex vivo* system may overcome even the limitations of the engineered tissue. In recent years human *ex vivo* lung tissue infections with various microorganisms, including pneumococci (Szymanski et al., 2012; Fatykhova et al., 2015), *Bacillus anthracis* (Chakrabarty et al., 2007), *Haemophilus influenzae* (Zhang et al., 2016), and IAV (Nicholls et al., 2007; Chan et al., 2009), were performed. In the human setting, most of the work focused on tropism, severity of infections, release of inflammatory mediators, and replication rates of the microorganisms. In addition, recently also experiments on swine influenza virus (SIV) and *S. suis* co-infections of the porcine *ex vivo* lung slices were reported. Meng and colleagues showed that SIV promotes subsequent bacterial infections in a two-step process of which the first initial step was dependent on capsule expression, whereas the second step of bacterial invasion into deeper layers was capsule-independent and required virus-mediated damage (Meng et al., 2015). However, this is just a beginning and more investigations are needed to unravel the complexity underlying these highly invasive infections.

In summary, bacterial and viral co-infections of the respiratory tract are highly lethal and present a dramatic burden for the global health system. The synergy between bacterial and viral infectious agents is related to a variety of factors, including epithelial barrier damage, exaggerated innate immune response, and cytokine storm. Despite many advances in recent years, more knowledge on mechanisms and immunology of disease progression is needed. The synergistic mechanisms between viruses and bacteria leading to enhanced morbidity and mortality are poorly understood. *In vivo* characterizations of these severe infections are mainly performed in mice which poorly resemble the human physiology and immune system. Several efforts have been made to establish other models, including ferrets, guinea pigs, rabbits, rats, and non-human primates. However, all have limitations. Here, we suggest using the porcine model, which provides obvious advantages in studies of human infectious diseases and should be considered much more frequent for future studies on severe infectious diseases, including pneumonia.

## Author Contributions

NS conceived the concept for this review article. NS and SO-H wrote the manuscript. NS, SO-H, TCM, BK, PV-W, and SH read, edited, and reviewed the manuscript.

## Conflict of Interest Statement

The authors declare that the research was conducted in the absence of any commercial or financial relationships that could be construed as a potential conflict of interest.
